# Mapping Geographic Disparities in Healthcare Access Barriers Among Married Women in Pakistan: Evidence from a Nationally Representative Survey

**DOI:** 10.3390/healthcare13192448

**Published:** 2025-09-26

**Authors:** Asifa Kamal, Gulzar H. Shah, Afrah Hafeez, Maryam Siddiqa, Charles Owens

**Affiliations:** 1 Department of Statistics, Lahore College for Women University, Lahore 54000, Pakistan; asifa.kamal@lcwu.edu.pk (A.K.); afrah.hafeez@usa.edu.pk (A.H.); 2 Jiann-Ping Hsu College of Public Health, Georgia Southern University, Statesboro, GA 30460, USA; cowens@georgiasouthern.edu; 3 Department of Mathematics & Statistics, International Islamic University Islamabad, Islamabad 44000, Pakistan; maryam.siddiqa@iiu.edu.pk

**Keywords:** barriers to care, Demographic and Health Survey (DHS), geospatial disparities Geographically Weighted Regression, healthcare access, health inequalities, health policy, spatial analysis, maternal health, Pakistan, women’s health, barriers to care

## Abstract

Healthcare access is a fundamental human right, yet barriers often negatively impact health, particularly in developing countries like Pakistan, where maternal mortality remains a crisis. This study aimed to identify factors influencing healthcare access barriers among married women aged 15–49 years using spatial analysis. **Methods:** Data were drawn from the 2017-18 Pakistan Demographic and Health Survey (PDHS), which included an unweighted sample of 8127 women. Healthcare access barriers were identified as the outcome variable. **Results:** A spatial analysis using ArcGIS 10.7.1 and SaTScan identified clustered distributions, with concentration areas identified in Gilgit Baltistan, Khyber Pakhtunkhwa (KPK), Federally Administered Tribal Areas (FATA), Punjab, and Balochistan. SaTScan highlighted primary clusters in FATA, Southern KPK, Northern Balochistan, and Eastern Punjab. Geographically Weighted Regression identified women who had five or more living children, respondents who did not have four or more antenatal care (ANC) visits, respondents who experienced a lower income (wealth index), respondents who did not participate in decision-making, respondents with a primary education, and respondents who accepted domestic violence as the significant predictors of healthcare access barriers. **Conclusions:** To improve women’s healthcare access, integrated policy interventions are needed, addressing socioeconomic disparities, strengthening national health policies, empowering women, and expanding healthcare accessibility. Strengthening health insurance and economic empowerment is crucial for achieving Sustainable Development Goals.

## 1. Introduction

Inadequacies and inequities in the allocation of healthcare and public health services for maternal and child health (MCH) elevate the risk of poor health outcomes in developing countries such as Pakistan [1]. Poor accessibility, affordability, and quality of health services are primary drivers of a myriad of maternal health issues, including maternal and neonatal mortality, complications during pregnancy and childbirth, anemia, and prenatal and postpartum depression [2]. Access to timely, equitable, and high-quality healthcare is essential for maintaining good health status and preventing disability and premature mortality [3]. Geospatial data visualization can effectively aid in identifying disparities in healthcare [3]. Sustainable Development Goals (SDG 3.1, SDG 3.7, and SDG 3.8) can be achieved through the improved access to basic medical care and universal access to affordable, high-quality essential medicines and immunizations [4,5].

Maternal and child health outcomes are worsened due to inequities in care and are exacerbated by Social Determinants of Health (SDoH) in Pakistan [6]. The maternal mortality rate (MMR) of 154 deaths per 100,000 newborns in Pakistan exceeds 430 maternal deaths per 100,000 live births in low-income countries [7]. The high MMRs in low-income countries like Pakistan are primarily attributable to the dire shortage of skilled birth attendants (SBAs) during deliveries, the limited number of healthcare facilities for deliveries, postnatal care (PNC), and antenatal care (ANC) [8,9]. Adverse MCH influences, including maternal and neonatal mortality, complications during pregnancy and childbirth, anemia, and prenatal and postpartum depression, remarkably influence maternal and infant well-being. Limited access to skilled birthing attendants (SBAs), antenatal care (ANC), postnatal care (PNC), and essential healthcare facilities further exacerbate these risks. According to the World Health Organization (WHO), maternal mortality rates in LICs exceed 430 deaths per 100,000 live births, a stark contrast to 12 per 100,000 in high-income countries. However, beyond maternal deaths, poor healthcare access leads to life-threatening complications during pregnancy and childbirth, contributing to neonatal mortality and long-term health consequences for mothers and infants alike [8,9]. Addressing these challenges requires a comprehensive approach to improving maternal and child healthcare services, ensuring equitable access to life-saving interventions, and strengthening healthcare systems in resource-limited settings.

Healthcare access barriers (HCBs) can be broadly classified into three categories—screening delays, absence of treatment, and healthcare inequalities—according to the healthcare access barrier model [10,11,12,13,14]. Obstacles in accessing healthcare services create a situation in which people’s health needs are not fully met or there is a lack of treatment, resulting in financial stress and unnecessary hospitalization [15]. The SDoH-related barriers, such as transportation obstacles, limited access to service locations, and a remarkable distance to medical facilities, are problematic in low-resource countries with poor infrastructure [16,17,18,19]. Living in close proximity to quality healthcare facilities has a remarkably positive influence on reducing maternal mortality, stillbirths, and infant mortality [6]. Women who live a substantial distance from medical services are generally unable to utilize them or reach them in a timely manner [20,21]. According to the World Health Organization (WHO), governments have a degree of responsibility for providing access to quality health services, as this is a fundamental human right of every individual [22,23]. Women-specific healthcare treatments, like adequate prenatal and postnatal care, reproductive healthcare, breast cancer therapy, and fistula treatment, raise challenges for them if there are barriers to healthcare accessibility. In Pakistan, 85% of women in urban areas and 66% of women in rural areas believe that getting to the health facility is a challenge for them [24].

Numerous challenges facing Pakistan’s healthcare system prevent access to offering women adequate and effective medical care, including a lack of financial resources, as Pakistan spends 38 US dollars (USD) per person on healthcare [25]. This level of resource allocation is remarkably low, even when compared to other developing countries, such as Ghana (USD 57 per person), the Philippines (USD 165 per person), and USD 85 per person in India [25]. Pakistan’s overall health budget is insufficient to fulfill the demands of the rapidly increasing population [26]. Despite rapid population growth, the increase in the healthcare budget was only 0.1%. Approximately 78% of Pakistanis bore their medical expenses by themselves [27]. Disparities in healthcare facilities can be observed all over the country, particularly between urban and rural areas [28]. In Pakistan, the availability of healthcare services varies significantly between the rich and the poor [29]. Over 50 million Pakistanis lack access to essential healthcare due to poverty and inadequate infrastructure [30]. Women face several hurdles to healthcare access, including limited decision-making power, home care responsibilities, restrictions on travel, and less priority given to women’s health within households [31].

Financial constraints, unemployment, low educational attainment, and rural residence are also significant barriers that limit healthcare access for women of reproductive age. Economic dependency on male family members restricts women’s ability to afford healthcare services, including consultation fees, medicines, and transportation costs. Unemployment further compounds these financial challenges, forcing women to rely on household income that may not prioritize their health and well-being. Low levels of education hinder women’s awareness of available healthcare services and their understanding of reproductive and maternal health, leading to delays in seeking medical attention [32,33]. Higher education improves healthcare literacy and reduces financial constraints, helping better-educated women mitigate access barriers [34,35,36]. Due to the scarcity of resources and large family sizes, women with high parity experience challenges in managing available health resources [37]. The affordability of healthcare is a significant issue, particularly in low-resource settings, such as low- and middle-income countries, and health insurance is one of the solutions [33,38]. Gender disparity in accessing healthcare worsens in the low socioeconomic segments of society, because poor households face more difficulties in accessing healthcare compared to women from affluent households [39,40]. Women’s decision autonomy enables them to better access healthcare despite social stigma and domestic violence. These characteristics contribute to increased abuse, and women are reluctant to report it, affecting their access to needed care [41,42]. Visiting a health facility within the last 12 months is used as a proxy for the recent utilization of healthcare services because it is linked to the exposure to healthcare [43,44,45]. Additionally, women living in rural areas face geographical barriers such as long distances to health facilities, poor road infrastructure, and a lack of transportation, making it difficult for them to access timely and quality healthcare services [46,47,48].

Situated in the north-west region of South Asia, Pakistan’s population is spatially diverse [49]. Given the diversity across these regions, detecting spatial disparities in healthcare access is imperative. Spatial analyses are increasingly recognized as an effective tool for guiding healthcare and public health interventions aimed at eliminating inequitable access [50]. However, studies utilizing those analytical tools are rare in Pakistan, although globally, spatial data visualization is common for revealing regional disparities in accessing healthcare [34,50]. The current study addresses this research gap through geospatial analyses of healthcare access challenges faced by women in Pakistan. Spatial analysis is more useful compared to conventional regression in the sense that conventional regression identifies why it occurs, while spatial regression also identifies where it occurs and its impact in that particular area [51]. Conventional regression offers a holistic approach, whereas Geographically Weighted Regression provides targeted solutions in hotspot areas. GWR probes further and explores relationships between predictors and the response variable that may have varying impacts at different locations [52,53]. It would be more helpful to prioritize resource allocation at various locations based on local patterns. The study provides empirical evidence for policy and practice, highlighting evidence-based strategies for allocating healthcare and public health resources to low-resource areas in an equitable manner.

## 2. Materials and Methods

This research utilized secondary data from the PDHS (2017-18), which was conducted by the National Institute of Population Studies (NIPS) under the authority of the Ministry of National Health Services. The ICF International coordinates PDHS surveys worldwide in collaboration with the United States Agency for International Development (USAID), the United Nations Population Fund (UNFPA), and the Department for International Development (DFID). The PDHS used a two-stage stratified sampling design. The survey’s first stage involved selecting 580 clusters comprising enumeration blocks (EBs), using a probability proportional to the population size in each EB. In the second stage, 16,240 households were selected from 561 clusters using systematic sampling. The survey was completed on 561 clusters, with 19 clusters excluded due to security concerns, resulting in a total of 15,671 households [54].

Data was acquired from the DHS Measure website after seeking approval (https://dhsprogram.com/data/dataset_admin/index.cfm (accessed on 25 August 2023)). The women’s file (Pakistan Demographic and Health Survey 2017 [PKIR71.DTA]. PKIR71.DTA.Rockville, Maryland, National Institute of Population Studies (NIPS), Islamabad, Pakistan, and ICF [54] was retrieved from the site to extract data on healthcare access barriers among women for analysis. Spatial data has been accessed from ICF. Utilizing a geographical coordinate file, cluster data were organized from 561 clusters or enumeration blocks (EBs). In the geographic coordinate data file, the 535th enumeration block was recorded as 0, so this EB was dropped from the study [55]. The women’s record file PKIR from 2017 to 18 PDHS was utilized; the total sample consisted of 15,068 women. The sample was restricted to married women because women’s empowerment indicators were only available for married women. The variables related to the decision-making power of women were also not available for unmarried women. After excluding records with missing data on independent variables/factors, such as ANC visits (43.6%), place of delivery (43.6%), decision-making power of a woman (3.8%), husband’s education (0.3%), and media exposure (0.1%), the weighted sample was reduced to 8127 women. The proportion of healthcare access barriers for clusters was computed by assigning weights. Similarly, the weighted proportion of independent variables was computed and used for spatial analysis to establish the representatives. These proportions were further used for spatial analysis [38]. Since missing data were not part of the outcome variable, except for GWR, the rest of the spatial analysis was based solely on the outcome variable; it did not affect all the analyses carried out in the study. In GWR, missing data compromised the statistical power; however, the use of complete case analysis preserved the integrity, as it was based on actual responses.

### 2.1. Outcome Variable

The dependent variables in this study are the barriers to women’s access to healthcare services. Four questions measuring access to healthcare services were posed to all women in the PDHS (2017-18): Women were asked whether each of the following factors is a big problem in seeking medical advice or treatment for themselves when they are sick: (a) “getting permission to go to the doctor,” (b) “getting money for advice or treatment,” (c) “distance to a health facility,” and (d) “not wanting to go alone.” Women who experienced at least one of above stated problems were classified as “having a barrier in accessing healthcare,” (coded 1) whereas women who had not experienced issues in any of those domains were classified as “having no problem” (coded 0) [34,36,43,48,56]. This dual method aims to recognize anyone who faces barriers to healthcare. Care can be delayed by or may not happen at all due to just one barrier. Hence, the composite measure draws out the population segment languishing with basic access, which is vital to the strengthening of the health system. To accommodate the complex survey design of the PDHS 2017-18 survey, we used the individual statistical weight variable (v005) and spatial weight variable (sv005). The indicators included in the four items of the survey constituted the dependent variable with no missing values in our analytic dataset (N = 15,068). Consequently, no imputation was made.

### 2.2. Independent Variable

The individual-level characteristics in the study included socio-demographic variables, such as the wealth index, age of the woman, respondent’s education, number of children, husband’s occupation, the woman’s work status, and husband’s education. The response categories for all independent variables are shown in Table 1. The variables reflecting gender dynamics included the attitude towards justifying wife-beating, decision-making power of a woman, woman’s ownership of assets, media exposure, and gender of household head. Access to care and other potential barriers to care included visiting a health facility in the last 12 months, health insurance coverage, place of delivery, whether termination of at least one pregnancy occurred, and having 4 or more antenatal care (ANC) visits. Two community-level factors, region and place of residence, were also included.

### 2.3. Data Management

The data were weighted using sampling weights, primary sampling units, and strata before any statistical analysis to restore the representativeness of the survey by considering the sampling design when calculating standard errors to obtain reliable statistical estimates. STATA 15 (StataCorp LLC, College Station, TX, USA) and SPSS version 25 (IBM Corporation, Armonk, NY, USA) were used for descriptive analysis. The ArcGIS Map 10.7.1 program (Esri, Redlands, CA, USA) was used to find statistically significant locations associated with healthcare access barriers among women in Pakistan. Kulldroff’s SatScan version 9.6 software (Martin Kulldorff, Harvard Medical School, Boston, MA, USA) was used to perform the statistical analysis of the spatial scan. Additionally, geographical analysis was utilized in the GWR4.09 software, (Centre for Advanced Spatial Analysis, University College London, London, UK).

### 2.4. Spatial Analysis

#### 2.4.1. Spatial Autocorrelation

The spatial autocorrelation (Moran’s I) was used to examine the barriers to women’s healthcare access and determine whether they were randomly distributed, scattered, or clustered. Global Moran’s I is a geographic statistic that converts the whole dataset into a single output value between −1 and +1. This allows us to measure spatial autocorrelation. Moran’s I near −1 shows that healthcare access barriers are widespread. Moran’s I near +1 implies that healthcare access barriers are clustered. Moran’s I near 0 indicates that healthcare access barriers are randomly distributed. A *p*-value of ≤0.05 was used to indicate the statistical significance of z-scores, suggesting the existence of a cluster. Moran’s I with *p* > 0.05, showing statistical non-significance, meant that observations were dispersed randomly [34]. Although spatial autocorrelation is usually performed using polygon data, in our case, cluster-level point data of the PDHS (201718) marking the centroid of sampled communities is used [57].

#### 2.4.2. Local Moran’s I

Local Moran’s I is a statistical method for identifying positively related clusters (both High-High, i.e., HH, and Low-Low, i.e., LL) and negatively related outliers (HL, i.e., High-Low, and LH, i.e., Low-High) of healthcare access barriers. A higher value is said to be an outlier if lower values mainly surround it, and a lower value is said to be an outlier if it is surrounded predominantly by higher values. A positive “I” value means that participation in adjacent cases is of identical values, whereas a negative “I” value suggests an outlier, i.e., a value is surrounded by dissimilar values [58].

#### 2.4.3. Getis-Ord-Gi* Analysis

The Getis-Ord Gi* statistic was used to detect significant hotspot and cold spot areas of healthcare access barriers. This helps to identify hotspot areas, indicating where these barriers are clustered [59]. To test the statistical significance of clustering, generate Z-scores and *p*-values. A Z-score between −1.96 and +1.96 and a *p*-value less than 0.05 indicate significant differences at the 95% confidence level. With a small *p*-value, a higher significant Gi-score indicates hotspot areas, while a smaller Gi-score indicates cold spot areas.

#### 2.4.4. Spatial Scan Analysis

The Bernoulli-based spatial SaTScan statistical analysis was used to examine the presence of statistically significant primary and secondary clusters of healthcare access barriers using Kulldorff’s SaTScan version 9.6 software. The SaTScan study builds upon insights gained from the initial Getis-Ord analysis, which demonstrates robustness in pinpointing hotspot locations and offers increased validation. The circular scanning window was used to identify the significant clusters of healthcare access barriers. Women who had faced barriers to accessing healthcare were used as cases, and those who had not encountered any barriers served as controls. The default upper limit of 50% of the population was chosen in the analysis because it allows for the detection of both small and large clusters of healthcare access barriers among women. A high likelihood ratio value and a small *p*-value identify the significant cluster with high healthcare access barrier rates.

#### 2.4.5. Spatial Interpolation

The kriging spatial interpolation technique was employed to estimate the prevalence of healthcare access barriers among women of unsampled areas, using sampled values as initial reference points. The technique enabled us to extrapolate between the data points of our samples (DHS clusters), determining values between points and generating a surface map. The essence of this interpolation was to visually portray the overall spatial trends and variations in healthcare access barriers, which would complement our statistical analyses, as it would give us a geographical representation of where the dependent variable is distributed. Various geostatistical interpolation methods exist, including ordinary kriging, simple kriging, and empirical Bayesian kriging, as described by [60,61,62]. Ordinary kriging and empirical Bayesian kriging are regarded as top alternatives, owing to their capacity to account for spatial autocorrelation and maximize weight allocation [34,63,64]. An empirical Bayesian kriging spatial interpolation method was developed for predicting healthcare access barriers in unsampled areas of Pakistan following cross-validation.

### 2.5. Geographically Weighted Regression (GWR)

Geographically Weighted Regression was employed to identify factors influencing the observed spatial distribution of healthcare access barriers among women. To accurately model the spatial relationship between variables, it is crucial to account for non-stationarity, acknowledging that the link between variables changes across locations within the study region. This approach addresses the challenge of non-stationarity by calculating local parameters that vary across different locations [65]. The two types of Geographically Weighted Regressions that were used were local and global. To examine the spatially varied relationships between factors and women’s healthcare access, we employed Geographically Weighted Regression (GWR) over Ordinary Least Squares (OLS). GWR accounts for spatial non-stationarity, where predictor effects differ across locations, which OLS (assuming global relationships) cannot capture. GWR fits local regression equations for each data point, allowing coefficients to vary geographically. This is crucial for policy, as it reveals unique local insights and disparities, unlike a “one-size-fits-all” OLS approach. ArcGIS was used to provide a visual representation of the spatial behavior of predictors throughout Pakistan.

## 3. Results

**Figure 1:**
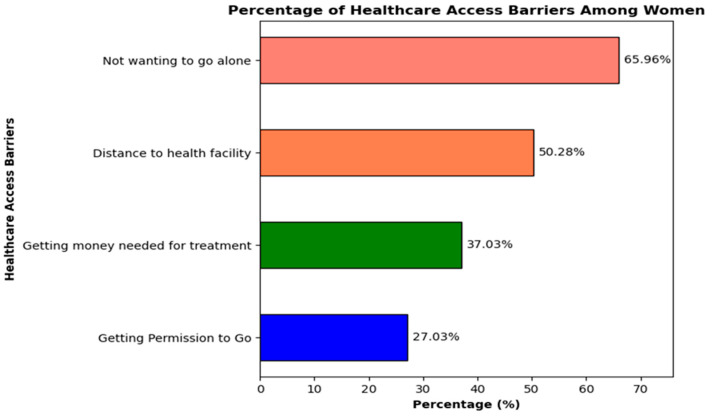
Percentage of healthcare access barriers among women aged 15–49 years.

Four questions regarding healthcare access barriers were used to compute the response variable for this study. The resulting response variable was then coded as 0 if the sum was 0, indicating that the respondent had never experienced any healthcare access barriers, and as 1 if any of the questions had a “yes” response, suggesting that women had experienced at least one healthcare access barrier. The response variable is titled “Healthcare Access Barriers Among Women”. The percentage of women aged 15–49 years who reported facing problems regarding healthcare access barriers is shown in Figure 1. The significant barriers these women reported were not wanting to go alone (65.96%) and the distance to the health facility (50.28%).

### 3.1. Percentage Distribution of Characteristics of Respondents

Table 1 showed that the age group in which a large proportion of women fall was observed between 20 and 34 years, accounting for 72.93% of the women; this is followed by the age group of 35–49 years, which constituted 23.21% of the sample; and the age group of 15 to 49 years made up 3.85% of the total. In terms of educational attainment, half of the women had not received any formal education, while the remaining women had attained primary education, 13.34% had secondary education, and 21.06% had higher education, accounting for 15.23%. The distribution of women who had undergone four or more antenatal care visits was almost equal, with 49.16% having visited for ANC and 50.84% not having visited for ANC.

#### 3.1.1. Global Spatial Autocorrelation (Global Moran’s I) of Healthcare Access Barriers

**Figure 2:**
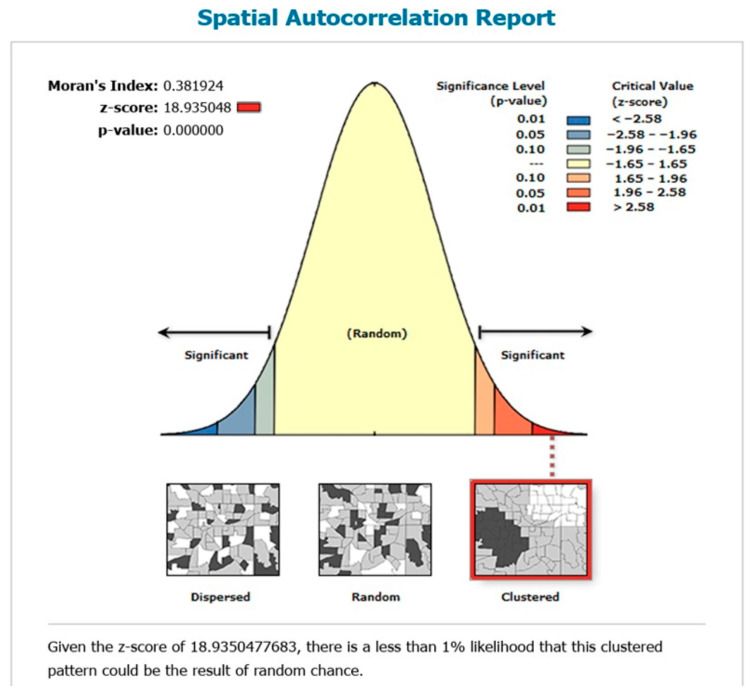
Spatial autocorrelation of healthcare access barriers among women in Pakistan; and map output: own analysis on ArcGIS.

The global spatial autocorrelation value of 0.3819 was observed, indicating that the distribution of healthcare access barriers among women aged 15–49 in Pakistan tends to exhibit clustering (Figure 2). The *p*-value associated with the global Moran’s I suggested that the distribution of healthcare access barriers among women varied significantly across the country.

#### 3.1.2. Local Moran’s I Analysis of Healthcare Access Barriers

The Local Moran’s I analysis was employed to identify clusters (High-High or Low-Low) and outliers (High-Low or Low-High) associated with healthcare access barriers among women in Pakistan. The results unveiled significant clusters and outliers within the study area. High-Low outliers (HL) represented clusters with a high proportion of healthcare access barriers among women, surrounded by low proportions of such barriers. On the other hand, Low-High outliers (LH) denote clusters with low proportions of healthcare access barriers among women, surrounded by a high proportion of these barriers.

**Figure 3:**
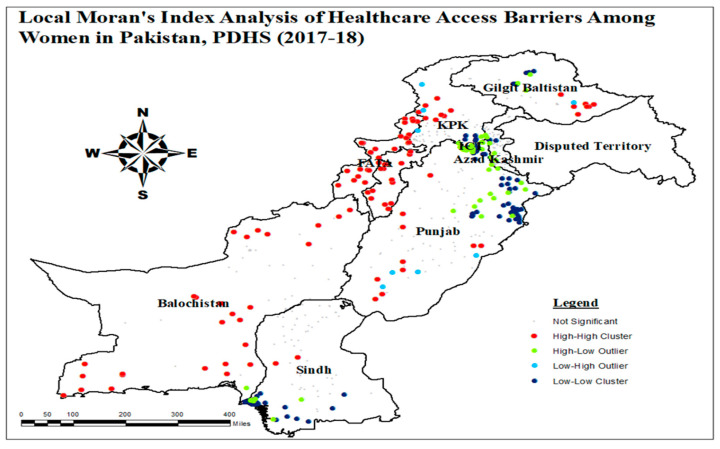
Clusters and outliers analysis of healthcare access barriers among women; and map output: own analysis on ArcGIS.

In Figure 3, locations were identified for analyzing clusters and outliers. Insignificant points, depicted in gray, had low z-values. Bright red points denoted High-High clusters, indicating that KPK, FATA, Balochistan, South Gilgit Baltistan, North-West Sindh, and Punjab exhibited closely grouped high values, signifying a higher risk of healthcare access barriers among women in these regions. Conversely, areas with a low prevalence of healthcare access barriers among women were represented by sky blue points, indicating Low-Low clusters in East-North Punjab, North Gilgit Baltistan, South Sindh, and ICT.

#### 3.1.3. Getis-Ord-Gi* Analysis of Healthcare Access Barriers

**Figure 4:**
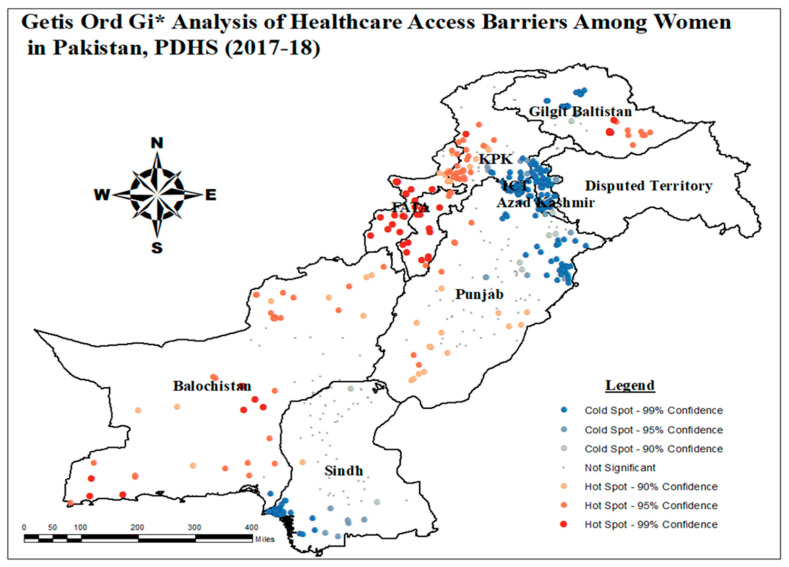
Hotspot analysis of healthcare access barriers among women aged 15–49 years; and map output: own analysis on ArcGIS.

The hotspot analysis determines the location of statistically significant geographic hotspot and cold spot clusters. It identified hotspot areas as clusters with a high percentage of healthcare access barriers and cold spots as clusters with a low percentage of healthcare access barriers. To determine whether a given location belonged to a hotspot or cold spot cluster, a Gi* value was computed. In Figure 4, hotspot clusters with a 99% confidence level represented regions with the highest prevalence of healthcare access barriers for women.

With a confidence level of 99%, hotspot areas were identified in FATA, Eastern Balochistan, and Eastern Gilgit Baltistan. Regions such as Punjab, Khyber Pakhtunkhwa (KPK), Balochistan, and Eastern Gilgit Baltistan were considered to be hotspots with a confidence level of 95%. In general, FATA and Balochistan had the highest concentration of hotspot locations, where most women face healthcare access barriers. Cold spot locations were characterized by a significant negative low z-score value, indicating a low occurrence of healthcare access barriers among women. Notable cold spot zones were observed in ICT, Azad Kashmir, Southern Sindh, Northern Gilgit Baltistan, and Northern Punjab.

#### 3.1.4. Spatial Scan Statistical Analysis of Healthcare Access Barriers

Compared to other hotspot analyses, the spatial scan statistical analysis provided a higher level of capability in detecting clusters of hotspots. The spatial scan statistics utilized a circular scanning window that traverses the entirety of the research area. To establish the Bernoulli model and determine the most significant barriers to healthcare access among clusters of women, those women who encountered obstacles to accessing healthcare were designated as cases, while those who did not were classified as controls.

**Figure 5:**
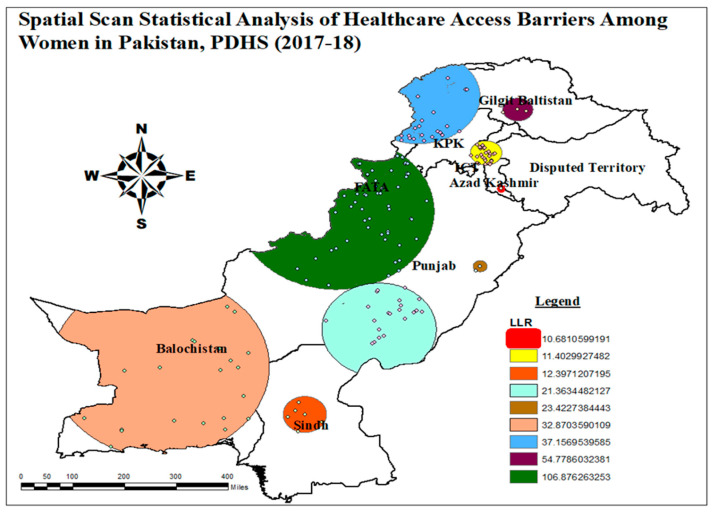
Satscan analysis of healthcare access barriers among women in Pakistan, PDHS (2017-18); and map output: own analysis on ArcGIS.

The primary cluster is represented by the first row of Table A1. These were the high prevalence of healthcare access barriers clusters, with a radius of 257.96 km and longitude and latitude of 32.423732 N, 69.429786 E. Women residing within the primary cluster area were found to have a 1.34 times higher probability of encountering barriers to healthcare access. The primary clusters, depicted in green in Figure 5, were found in regions such as FATA, Southern KPK, Northern Balochistan, and Eastern Punjab. Table A1 illustrates that the LLR of healthcare access hurdles was highest for FATA, Southern KPK, Northern Balochistan, and Eastern Punjab, with a value of around 106.87.

Figure 5 illustrates the locations of the four secondary clusters, which are situated in the Gilgit Baltistan region, as indicated by the purple color. Additionally, 22 secondary clusters were identified in northern FATA, KPK, and Western Gilgit Baltistan, denoted by the red color. Similarly, the Balochistan region revealed the presence of 22 clusters within the secondary cluster, represented by the blue color in Figure 5. Furthermore, additional secondary clusters were discovered in Punjab, Sindh, and Azad Kashmir.

**Figure A1:**
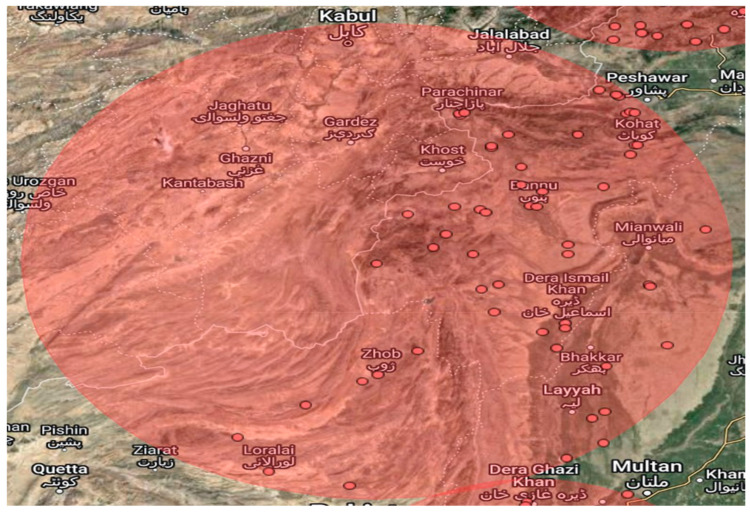
Satellite view of primary cluster of healthcare access barriers among women in Pakistan. Note: map output: own analysis on ArcGIS and red colour shows clusters with high proportion of women experiencing healthcare access barriers.

Satscan also provided a satellite view identifying the area with significant clusters and a high risk of healthcare access barriers. Primary clusters of healthcare access barriers were shown in Figure A1. These cities were located in FATA, Southern KPK, Northern Balochistan, and Eastern Punjab. Cities included were Parachinar, Dera Ismail Khan, Mianwali, Near Khosr, Bhakkar, Layyah, Zhob, and Loralai, experiencing clusters with a relative risk of 1.34 of healthcare access barriers in these areas (Table A1). Only one satellite view of primary clusters was mapped to avoid too many figures.

#### 3.1.5. Spatial Interpolation Technique of Healthcare Access Barriers

**Figure 6:**
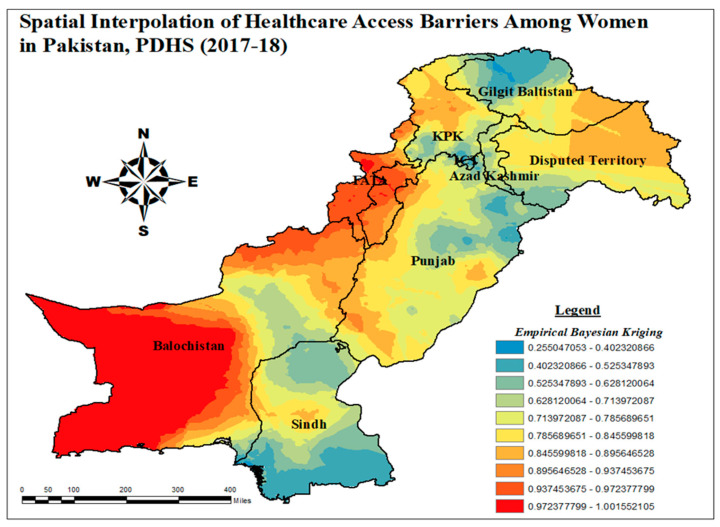
Empirical Bayesian kriging interpolation of healthcare access barriers among women in Pakistan; and map output: own analysis on ArcGIS.

Spatial interpolation was used to estimate the distribution of healthcare access barriers among women in unsampled areas of Pakistan, utilizing Estimated Bayesian (EB) models. The empirical Bayesian kriging method was identified as the most effective approach for finding the distribution of healthcare access barriers among women in Pakistan after comparison using cross-validation methods. Figure 6 illustrates the notable prevalence of obstacles to healthcare access encountered by women aged 15–49 years in Pakistan. In the majority of Balochistan and Northern FATA, there exists a likelihood of 97% to 100% that women may face hindrances in accessing healthcare. In specific regions, such as FATA and Balochistan, the percentage of women who experienced such barriers ranged from 93% to 97%. Similarly, in the areas of northern KPK and South-Eastern Punjab, there was a probability of 89% to 93% for women to encounter barriers to healthcare access. Conversely, the regions with the lowest risk were identified as Sindh, Northern Punjab, and Northern Gilgit Baltistan, where the likelihood of healthcare access barriers for women ranged from 25% to 40%.

### 3.2. Geographically Weighted Regression (GWR) of Healthcare Access Barriers

The determinants of the clustered arrangement of obstacles in accessing healthcare among women aged 15–49 in Pakistan were ascertained through the utilization of a Geographically Weighted Regression. Both global and local Geographically Weighted Regressions were carried to identify the factors that contribute to barriers in accessing healthcare among women, as well as the fluctuating relationship between these factors and healthcare access barriers.

#### 3.2.1. Global Geographically Weighted Regression (GGWR) of Healthcare Access Barriers

The GGWR, also known as Ordinary Least Squares, illuminated the predictors of healthcare access barriers among women, as well as whether a Local GWR fit was necessary based on assumptions.

Exploratory regression was fitted before performing a global Geographically Weighted Regression. Variables with a 60% or above significance level in the exploratory regression analysis were chosen for GGWR. The global GWR was carried out to identify the significant predictors of healthcare access barriers among women. A unit increase was seen in women who had five or more living children. Respondents who did not attend four or more ANC visits, poorer (wealth index), respondents who did not take part in decision-making, respondents’ primary education, and respondents who accepted domestic violence increased healthcare access barriers among women by 0.243200, 0.314446, 0.090962, 0.551590, 0.439152, and 0.167213, respectively.

All predictors had variances in the Inflation Factor (VIF) <7.5, which identified the absence of multicollinearity, as shown in (Table 2). Table 3 included diagnostic measures such as the Koenker (BP) statistics and Jarque–Bera statistics that were found to be significant, indicating the presence of non-stationarity and the biasedness of predictors (non-normal distribution of residuals) in the model, respectively. In the case of spatial non-stationarity and spatial heterogeneity, OLS is not appropriate and justified the application of GWR. GWR is also robust to mild non-normality, and it still can provide useful insight even when residuals are non-normal [53]. Researchers used GWR for non-normal data [66,67]. The adjusted R square was 0.9314, which is sufficient, as it showed 93% variability in healthcare access barriers among women, was explained by the model. Because the model had non-stationarity and biasedness, indicating a violation of assumptions, local Geographically Weighted Regression was performed.

**Figure 7:**
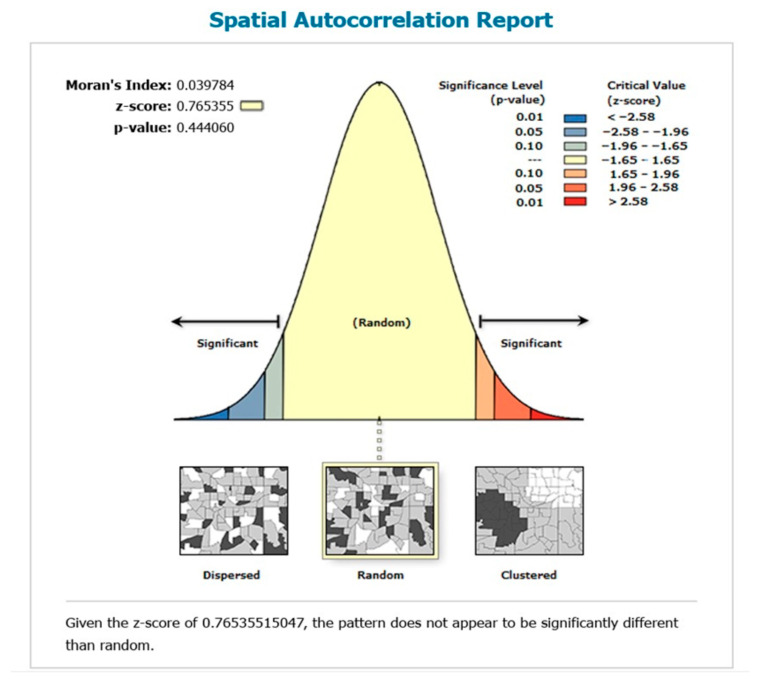
Spatial autocorrelation of residuals of GGWR; and map output: own analysis on ArcGIS.

Figure 7 demonstrates the spatial random distribution of the GWR residuals, and the non-significant *p*-value of 0.444 > 0.05 supported the acceptance of the null hypothesis that the distribution of residuals is random.

#### 3.2.2. Local Geographically Weighted Regression (GGWR) of Healthcare Access Barriers

The local Geographically Weighted Regression (LGWR) model was performed using GWR4 software to calculate the local coefficients of the variables and determine whether LGWR was better than the global regionally weighted regression (GGWR). The diagnosis of LGWR was listed in Table 4 with an AICc value of 2735.180, which was lower than the AICc value of GGWR (2881.818). Additionally, both R-squared and adjusted R-squared values were higher in the LGWR. This showed that the LGWR was more suitable than the GGWR. In the LGWR, the model explains a 96% variation in healthcare access barriers among women and has a lower AICc, supporting its superiority over GGWR.

**Figure A2:**
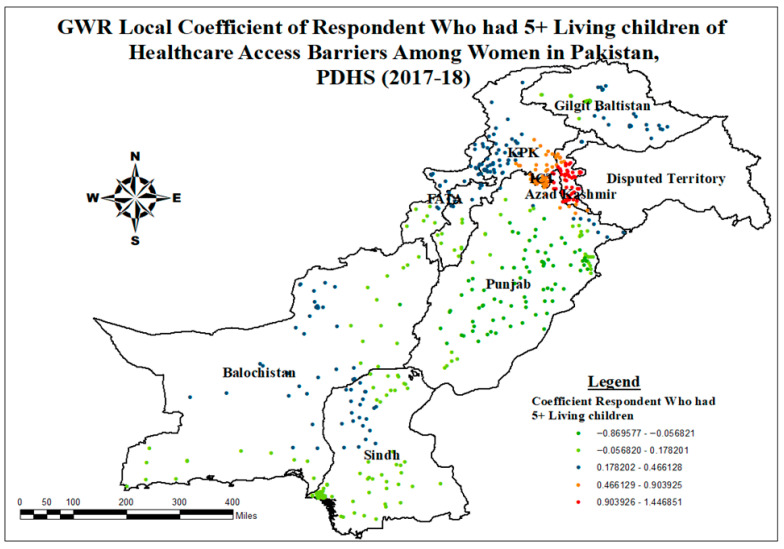
Varying effect of respondents who had 5+ living children on healthcare access barriers among women in Pakistan. Note: map output: own analysis on ArcGIS.

**Figure A3:**
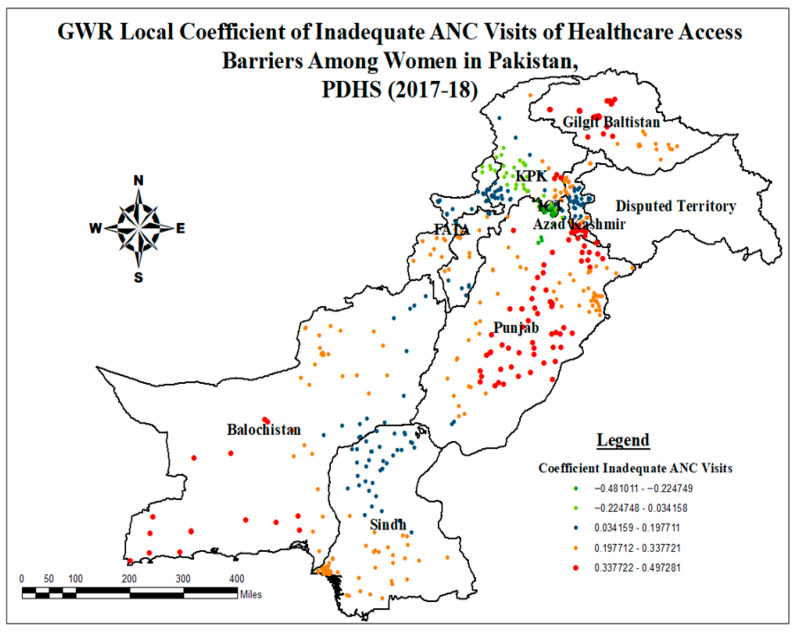
Varying effect of inadequate ANC visits on healthcare access barriers among women in Pakistan. Note: map output: own analysis on ArcGIS.

**Figure A4:**
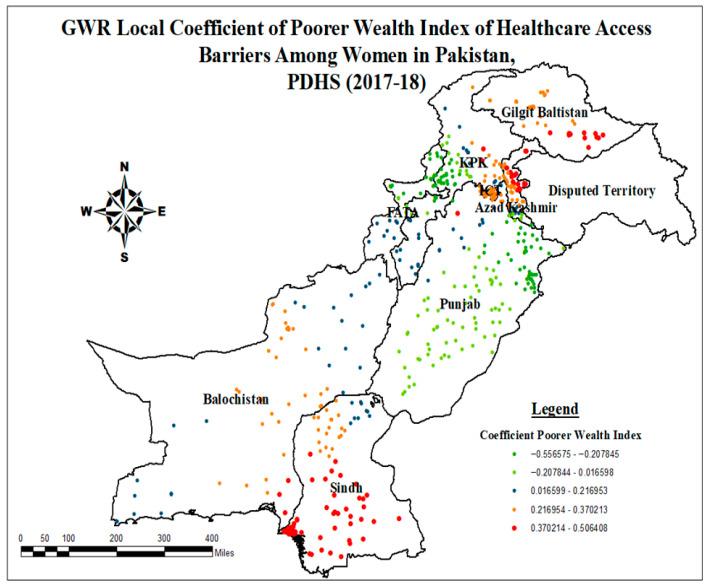
Varying effect of poorer wealth index on healthcare access barriers among women in Pakistan. Note: map output: own analysis on ArcGIS.

ArcGIS software was utilized to depict variations in the coefficients of predictors. In the graphical representation, Figure A2, regions with a notable positive impact of women having children ≥5 on healthcare access barriers were observed in Azad Kashmir, Islamabad Capital Territory (ICT), and Eastern Khyber Pakhtunkhwa (KPK). Figure A3 illustrates that the predictor “inadequate ANC Visits” was found to have a significant impact on healthcare access barriers among women in most of Punjab, Gilgit Baltistan, Northern Balochistan, Northern Azad Kashmir, and selected areas of KPK. The influence of a poorer wealth index on healthcare access barriers among women was prominent in specific regions, as shown in Figure A4, particularly in Southern and Eastern Gilgit Baltistan, Southern Sindh, Northern Azad Kashmir, Eastern KPK, and some parts of Balochistan.

**Figure A5:**
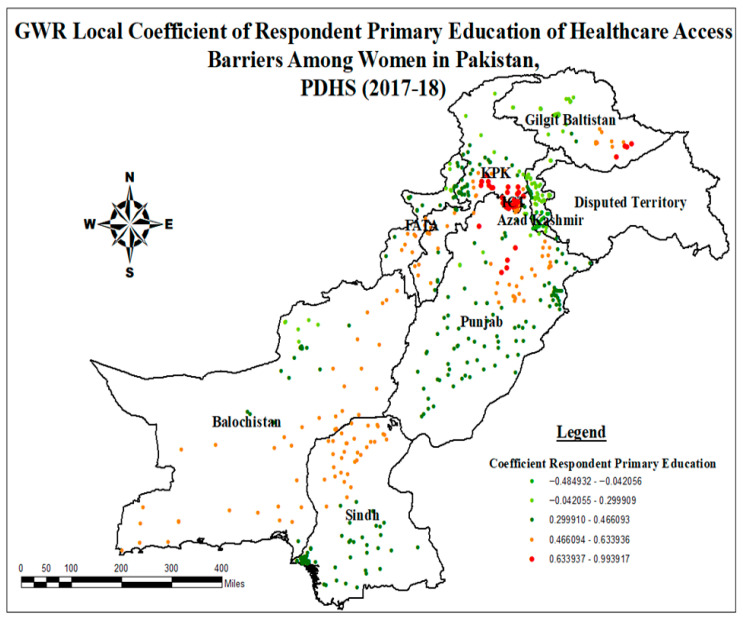
Varying effect of respondent’s primary education on healthcare access barriers among women in Pakistan. Note: map output: own analysis on ArcGIS.

**Figure A6:**
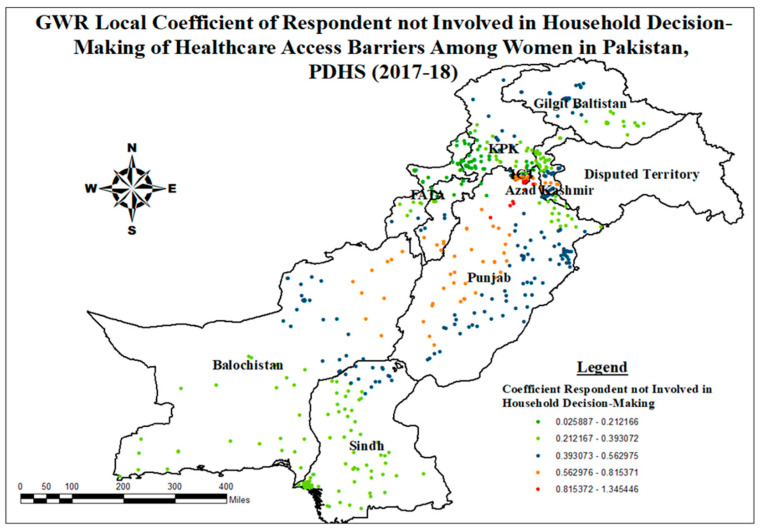
Varying effect of respondents not involved in household decision-making on healthcare access barriers among women in Pakistan. Note: map output: own analysis on ArcGIS.

**Figure A7:**
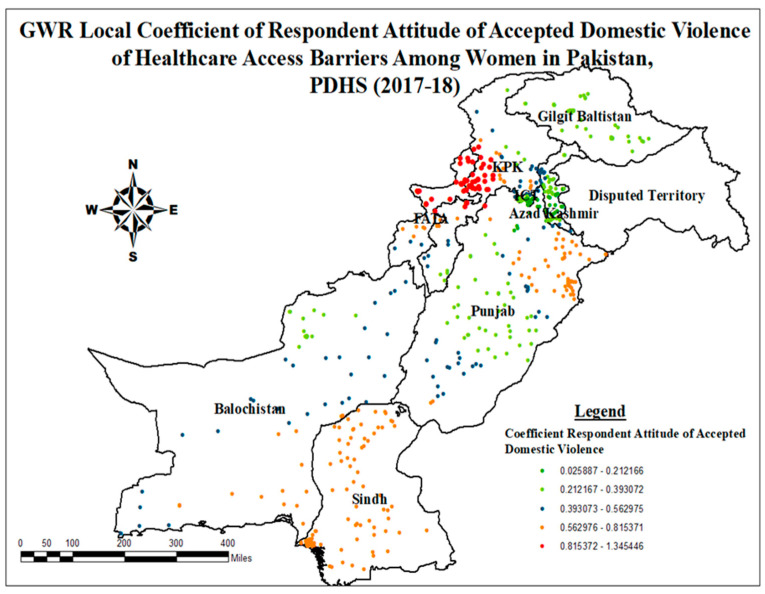
Varying effect of respondent’s attitude of acceptance toward domestic violence on healthcare access barriers among women in Pakistan; and map output: own analysis on ArcGIS. Note: map output: own analysis on ArcGIS.

Figure A5 depicts the positive impact of women with a primary education on healthcare access barriers, highlighting significant effects in ICT, Southern and Eastern KPK, Northern Punjab, and Eastern Gilgit Baltistan. Figure A6 demonstrated that women not involved in household decision-making had to face substantial barriers to healthcare access in Upper Punjab and ICT. Finally, Figure A7 indicates that the impact of women accepting behavior towards domestic violence showed a significant risk for healthcare access barriers in the majority of Southern and Western KPK and Northern Federally Administered Tribal Areas (FATA).

## 4. Discussion

This study identified the spatial distribution of healthcare access barriers among married women of reproductive age using the 2017-18 PDHS data. This study found that nearly two-thirds of married women of reproductive age faced healthcare access barriers due to at least one or more of the four primary reasons identified. The most commonly cited reasons were not wanting to go alone and the distance from health facilities. Then come financial constraints and permission restrictions. Pakistani women had restricted mobility due to social and cultural reasons. In Pakistan’s healthcare system, there are District Headquarters Hospitals (DHQs), Tehsil Headquarters Hospitals (THQs), Rural Health Centers (RHCs), and Basic Health Units (BHUs). Unfortunately, in RHCs and BHUs, there is a lack of staffing, particularly among health professionals who prefer to not be located in areas with limited health facilities. As a result, people move to DHQs and THQs to be near the healthcare facilities. These DHQs and THQs are located far from certain areas, creating a major hindrance for women to access healthcare. The rate of lower-middle-income poverty was approximately 42.3% for the financial year of 2024, revealing the unaffordability of healthcare for Pakistani people [68]. According to a study, Pakistan had a higher percentage of women facing barriers to healthcare access (72%) compared to Ghana (50%) [34], Benin (60.4%) [35], and Kenya (57%) [69]. This could be attributed to socioeconomic disparities across countries, which may also influence the obstacles to accessing healthcare [69].

Findings from the spatial analysis revealed that the distributions of healthcare access barriers for women in Pakistan were not random, indicating healthcare access disparities. Regional variations existed in the geographical distribution of these barriers. Significant hotspots of healthcare access barriers among women were identified in Gilgit Baltistan (GB), Khyber Pakhtunkhwa (KPK), Federally Administered Tribal Areas (FATA), Punjab, and Balochistan regions. One of the reasons is that Pakistan allocated only 1.2% of its GDP to the health sector in 2020–21 [25]. Geographical challenges, financial constraints, and socio-cultural restrictions hinder healthcare access in regions like FATA, KPK, and Gilgit Baltistan. These restrictions and challenges include difficulty in physical access to healthcare due to mountain terrains and a lack of permission for women to go alone [25,70,71,72]. These regions also had a strong patriarchal tradition. Women’s mobility is restricted in these areas due to tribal values. Women are allowed to travel only when accompanied by a male family member. The lack of women’s education and employment in these areas also limited female mobility. The SaTScan analysis identified primary clusters in FATA, Southern KPK, Northern Balochistan, and Eastern Punjab. In addition to this spatial interpolation, the highest magnitude of healthcare access problems was detected in Balochistan and FATA. The reasons behind this are limited healthcare facilities and security concerns. In particular, the areas like FATA and some areas of Balochistan and KPK have security issues hindering access to care due to women’s safety concerns [73,74]. The inequitable distribution of resources is one of the reasons that keep some regions deprived [25].

Geographically Weighted Regression identified women who had 5+ living children, respondents who did not attend 4+ ANC visits, respondents who were poorer (wealth index), respondents who did not take part in decision-making, respondents who had a primary education, and respondents who accepted domestic violence as significant predictors of healthcare access barriers. These predictors were contributing to the prevalence of healthcare access barriers in Pakistan. Healthcare insurance [34,38] and women’s ownership of assets [31] were the two predictors used in the literature as potential predictors of healthcare access barriers. Neither had a significant contribution to healthcare access barriers in the current study due to low variability and low statistical power, since these factors provided information at the exploratory stage, so they were retained in the study at that stage but did not affect the GWR.

The issue of healthcare access barriers among women in Pakistan was notable among women who had five or more living children. The results of this investigation are consistent with the findings of the Bangladeshi study [75]. Azad Jammu and Kashmir and ICT were found to have the highest GWR coefficients of the highest parity in this study. In these regions, the mean number of children born to women aged 40–49 was also high (KPK = 5.6, ICT = 3.9, and AJK = 5.1) [54]. The suburban areas of ICT, particularly the surrounding rural areas, are inhabited by economically less privileged communities, and they face hurdles to accessing healthcare amenities due to out-of-pocket health expenditure in cases of a large family size. The possible explanation might be that managing large families and several children at home, as well as the lack of resources associated with a large family size, are typical challenges faced by women with high parity [37].

This study revealed that women from low-income households faced higher barriers to accessing healthcare. Similar socioeconomic barriers have been observed across low-resource settings globally [69,76,77,78,79,80,81]. In terms of poverty, regional or geographical inequality has been observed in Pakistan. The southern part experienced poverty more compared to other regions. Differentials in poverty are also observed between urban, peri-urban, and rural areas. This polarization of poverty led to discrimination in the allocation of expenditures on welfare programs, particularly in education and health. Poverty also mainly effected the women’s intra-household opportunities and dispersal of resources [82]. One of the primary causes of the barriers to healthcare access was income; those with a low wealth index faced access problems. Women who belonged to higher wealth indices may find it easier to access medical treatment [74,80,83,84]. Furthermore, a family’s financial capacity frequently affects the accessibility of healthcare, since accessibility is adversely affected by both direct and indirect expenses, such as those associated with paying for treatments and medications, as well as transportation and unpaid labor [85].

Similarly, there was a statistically significant association between inadequate ANC visits and women who had reported healthcare barriers. This conclusion is consistent with research findings from studies conducted in Pakistan [45,85], Rwanda [86], and Nigeria [45,86]. The study highlighted that the GWR coefficients for inadequate ANC visits were higher in Northern Gilgit Baltistan, Southern Balochistan, Southern Azad Jammu Kashmir, Punjab, and certain districts of KPK. The main reasons are that rural and urban Pakistani women do not use the ANC services in public or private sectors due to the lack of finances, lack of accessibility issues to government health institutions due to long distance, restrictions imposed by their husbands or mothers-in-law, household work, inexperienced healthcare staff, and their busy working hours if a woman is engaged in economic activity [25,74,87]. The most common reason for pregnant Pakistani women to not access a health center for ANC treatment is restrictions by husbands [87].

It was found in the study that women’s primary education in Pakistan was a strong predictor of women facing barriers in accessing healthcare; this finding is consistent with other studies performed in Bangladesh [81], Pakistan [74,88], and Sub-Saharan Africa [40]. Highly educated women are more likely to hold well-paying positions, which can make it easier for them to afford healthcare, even if it is far away [88]. Additionally, education can also lead to stronger health literacy and awareness of fundamental human rights [74,83,88]. In the present study, employing education as an independent variable mainly proved that education influences employment and economic growth for the individual and the household [81,88,89,90]. This can be particularly helpful in removing barriers that hinder access to health services [91]. The higher education and employment of women could mitigate the challenges of cost, distance, and decision-making on health services [40].

Empowerment characteristics, such as decision-making autonomy, are critical for gaining access to healthcare services. According to our research, women who had less decision-making authority were more likely to encounter obstacles while trying to obtain healthcare, which is consistent with other studies’ findings in Brazil [92], Iran [93], India [94], and Afghanistan [95]. Healthcare hurdles were observed for women living in ICT and Punjab due to their limited decision-making authority at home. Decision-making, however, poses significant obstacles in Pakistan, because women are susceptible to having less authority over decisions that affect their health and well-being [96].

Domestic violence is rampant in Pakistan [97]. Our study showed that the attitude towards domestic abuse is a significant predictor of healthcare access issues. This study revealed that the women who had not accepted domestic violence had fewer chances of healthcare access issues compared to those who were receptive to it. This result is similar to other studies [98,99]. This study found that KPK and FATA had higher GWR coefficients, indicating an association between acceptance attitudes towards domestic violence and access to healthcare barriers. Lower education levels may make it difficult for victims of abuse to obtain the assistance they need and report abuse. Consequently, they accept domestic violation [100,101,102]. The other reason for this result could be that women who are victims of domestic violence from their male partners may find it physically challenging to access health facilities [103,104,105,106]. Combined with these issues, women are either less likely to inform others about violent situations or delay going to the doctor for a specific sickness, which then results in erroneous diagnoses, unnecessary treatment, or no treatment for the ailment in question [105]. A crisis center can only partially address the special difficulties that domestic abuse generates when it comes to receiving healthcare. To better meet the health requirements of women who experience domestic abuse, there has to be improved collaboration with the healthcare system and women’s protection centers for domestic violence [107].

## 5. Conclusions

This study found that two-thirds of married women encountered obstacles while attempting to access healthcare. It depicted disparities in healthcare access issues with the inequitable distribution of social determinants of maternal health as a potential “upstream” cause. Women in Gilgit Baltistan, Khyber Pakhtunkhwa (KPK), Federally Administered Tribal Areas (FATA), Punjab, and Balochistan faced more healthcare access barriers compared to those in Pakistan’s other regions. The significant predictors of healthcare access barriers in Pakistan are high fertility levels, indicated by five or more living children, being receptive to domestic violence, inadequacy of ANC visits, poorer wealth index, women’s primary education level, and lower level of decision-making power. The study suggests that an integrated policy intervention is essential to address women’s barriers to healthcare access. By addressing socioeconomic issues, strengthening national health policy, empowering women, and promoting flexible access to healthcare, Pakistan’s women’s health can be improved by focusing on the hotspot areas identified in the study. Special attention and resources should be allocated to areas with inadequate access to healthcare. Addressing Social Determinants of Health (SDoH), such as limited or lack of transportation, remote and difficult-to-access healthcare facilities, and other barriers, must be addressed to improve access to essential healthcare services and enhance health outcomes.

## 6. Limitations

This study utilized secondary data, which has inherent limitations. For example, the high number of missing data points made it difficult to determine factors such as whether the respondent earns more than her partner and who makes decisions about spending the respondent’s earnings. These variables may also contribute to women’s difficulties in accessing healthcare but were not included due to a high percentage of missing values. Further, the data were self-reported without any independent verification. To fully understand the factors affecting healthcare access in Pakistan, it is vital to identify and evaluate environmental, disease-related, and health system components that were not available in the survey. This will provide a more comprehensive understanding of the issue. It should also be noted that the kriging interpolation method assumes that the joint probability remains constant across the research region and that the space under study is stable. This means that the interpolated values may not accurately reflect the real values in areas that are not stable. Additionally, the SatScan software used in this study can only identify circular clusters and may not be able to detect irregular clusters. The factors of ANC visits and place of delivery included in the study had high missing observations that may have an impact on their statistical power.

## 7. Recommendations

The study requires further validation of the relationships found using data from other surveys that have information on healthcare access barriers, as well as employing multiple imputation methods to handle missing data within the same survey. The government of Pakistan should provide free healthcare services to facilitate access for women. The Lady Health Worker Program in Pakistan, for instance, has shown promise in providing maternal and child health services door-to-door, especially in rural areas that are underprivileged. In hotspot areas like FATA, Balochistan, and Gilgit Baltistan, this program might be a scalable outreach model. The government should provide healthcare access, especially to the poor and underprivileged sections of women with a low level of education, thereby facilitating access to vital health services, particularly maternal and reproductive healthcare, in the hotspot areas of FATA and in the provinces of Balochistan and Gilgit Baltistan (GB) on a priority basis. Additionally, launching a tailored media campaign can also help increase awareness around women’s health concerns. This will empower women to make knowledgeable choices regarding their health and life. In Pakistan’s conflict-affected regions, pilot crisis centers and NGO-led projects have also shown promise in helping women who are victims of domestic abuse and face obstacles to receiving healthcare.

The impact of the acceptance of domestic violence and lack of decision-making power in the healthcare access hotspot areas identifies the importance of remarkably increasing the awareness among women on their basic health rights with the help of the government and NGOs in the hotspot areas. The government can establish crisis centers to protect women from domestic violence. In FATA, Balochistan, and GB it is recommended to initiate a door-to-door health program, such as the Lady Health Visitors. These policy implications contribute to achieving SDG 3.1, SDG 3.7, and SDG 3.8.

## Figures and Tables

**Table 1:** Percentage distribution of characteristics of married women aged 15–49 years.

Women Characteristics	N (=8127)	Percentage
Individual-Level		
Age		
15–19	284	3.49
20–34	6028	74.17
35–49	1815	22.34
Women’s Educational Status		
No education	3763	46.30
Primary	1296	15.95
Secondary	1905	23.45
Higher	1163	14.31
4+ ANC Visits		
Inadequate ANC visits	4085	50.26
Adequate ANC visits	4042	49.74
Place of Delivery		
Home	2582	31.76
Health facility	5546	68.24
Media Exposure		
No	2975	36.61
Yes	5152	63.39
Currently Working Status		
No	7050	86.75
Yes	1077	13.25
Visited a Health facility in the Last 12 Months		
No	1566	19.27
Yes	6561	80.73
Health Insurance (HI) Coverage		
No HI	8000	98.44
Yes HI	127	1.56
Terminated Pregnancy (TP)		
No TP	5525	67.98
Yes TP	2602	32.02
Attitude Towards Domestic Violence		
Did not accept violence	4459	54.87
Accepted violence	3668	45.13
Relationship/Household-Level		
Gender of Household Head		
Male	7203	88.62
Female	924	11.38
No. of Living Children		
No children	88	1.08
1–2 children	3513	43.22
3–4 children	2662	32.75
5 or more children	1864	22.94
Husband’s Working Status		
Not working	251	3.09
Working	7876	96.91
Wealth Index		
Poorest	1801	22.17
Poorer	1789	22.02
Middle	1695	20.86
Richer	1482	18.23
Richest	1359	16.72
Husband’s Educational Status		
No education	2148	26.43
Primary	1277	15.72
Secondary	3033	37.32
Higher	1669	20.54
Women’s Assets of Ownership		
Does not own house/land	7887	97.05
Own house/land	240	2.95
Decision-Making Power of Women		
Women not involved	5471	67.32
Women involved	2656	32.68
Community-Level Factors		
Region		
Punjab	3359	41.33
Sindh	1550	19.07
KPK	1088	13.39
Balochistan	370	4.55
GB	660	8.12
ICT	52	0.64
AJK	894	11.01
FATA	154	1.90
Residence		
Urban	2429	29.89
Rural	5698	70.11

**Table 2:** Significant predictors of healthcare access barriers using Ordinary Least Squares (OLS).

Variables	Coefficients	*p*-Value	VIF
Intercept	0.32	0.070	-------
Women who have 5+ living children	0.24	0.000 *	3.45
Inadequate ANC visits	0.31	0.000 *	6.09
Poorer (wealth index)	0.09	0.020 *	2.19
Respondent did not take part in decision-making	0.55	0.000 *	2.06
Respondent’s primary education	0.443	0.000 *	4.84
Respondent accepted domestic violence	0.17	0.000 *	3.96

* *p*-value < 0.05, VIF = Variance Inflation factor tables footnote.

**Table 3:** Diagnosis for global Geographically Weighted Regression.

Diagnostic Measures	Value	*p*-Value
AICc	2881.82	------
R square	0.93	------
Adjusted R square	0.93	------
Koenker (BP) Statistics	109.56	0.000 *
Jarque–Bera Statistics	902.22	0.000 *

* *p*-value < 0.05.

**Table 4:** Diagnostic measures of LGWR.

Diagnostic Measures	Value
AICc	2735.18
R square	0.96
Adjusted R square	0.95

## Data Availability

Data has been accessed from measure DHS website after formal permission: https://dhsprogram.com/data/available-datasets.cfm (accessed on 25 August 2023).

## Abbreviations

AJK	Azad Jammu Kashmir
ANC	Antenatal Care
ANN	Artificial Neural Network
AUROC	Area Under the Receiver Operating Characteristic Curve
BHU	Basic Health Units
CV	Cross-Validation
DT	Decision Tree
EBK	Empirical Bayesian Kriging
FATA	Federally Administrated Tribal Areas
GB	Gilgit Baltistan
GGWR	Global Geographically Weighted Regression
GPS	Global Positioning System
GWR	Geographically Weighted Regression
ICT	Islamabad Capital Territory
KNN	k-Nearest Neighbors
KPK	Khyber Pakhtunkhwa
LGWR	Local Geographically Weighted Regression
LR	Logistic Regression
ML	Machine Learning
NB	Naïve Bayes
NHV	National Health Vision
NIPS	National Institute of Population Studies
OLS	Ordinary Least Square
PDHS	Pakistan Demographic and Health Survey
PHC	Primary Health Care
PSU	Primary Sampling Unit
RF	Random Forest
RHC	Rural Health Centers
SBA	Skilled Birth Attendants
SDG	Sustainable Development Goals
SMOTE	Synthetic Minority Oversampling Technique
SVM	Support Vector Machine
THQ	Tehsil Headquarter Hospitals
UHC	Universal Health Coverage
USA	United States of America
USAID	United Nations International Children
UTM	Universal Transverse Mercator
VIF	Variance Inflammation Factor
WHO	World Health Organization

## Appendix A

**Table A1:** Spatial scan statistical analysis results of healthcare access barriers among women in Pakistan PDHS (continued).

Enumeration Blocks	Coordinates/Radius	Relative Risk	Percentage of Cases in Area	LLR	*p*-Value
411, 412, 406, 408, 413, 83, 410, 84, 407, 409, 342, 79, 341, 75, 340, 74, 414, 76, 77, 78, 415, 72, 403, 404, 80, 81, 82, 338, 399, 401, 402, 400, 73, 111, 106, 405, 105, 339, 69, 70, 71, 107, 197, 198, 337, 98, 391, 336, 392, 395, 390, 397, 398, 393, 200, 201	(32.423732 N, 69.429786 E)/257.96 km	1.34	93.5	106.876263	0.000000 **
507, 474, 512, 511	(35.515992 N, 74.517198 E)/38.11 km	1.40	99.4	54.778603	0.000000 **
2, 4, 3, 1, 5, 6, 482, 12, 13, 481, 7, 384, 9, 387, 8, 388, 383, 10, 11, 17, 389, 14	(35.891914 N, 71.726873 E)/156.20 km	1.31	92.7	37.156954	0.000000 **
371, 370, 369, 368, 365, 356, 357, 367, 361, 364, 366, 378, 377, 373, 362, 360, 379, 358, 355, 372, 363, 380	(27.760806 N, 64.515493 E)/299.89 km	1.40	100.0	32.870359	0.000000 **
171, 170	(30.803986 N, 73.460618 E)/18.69 km	1.36	97.1	23.422738	0.000000 **
212, 211, 215, 195, 214, 213, 202, 199, 203, 194, 185, 180, 207, 182, 187, 193, 192, 204, 346, 186, 181	(28.897297 N, 70.636209 E)/155.68 km	1.18	83.3	21.363448	0.000001 **
315, 317, 236, 316, 318, 252	(26.349390 N, 68.560090 E)/60.27 km	1.25	89.1	12.397121	0.002914 *
522, 517, 532, 516, 529, 519, 523, 518, 524, 534, 531, 520, 538, 521, 530, 515, 539, 26, 525, 537, 536, 22, 546, 553, 28, 550, 549, 90, 94	(34.211114 N, 73.629460 E)/40.62 km	1.16	82.6	11.402993	0.010000 *
558	(33.131774 N, 74.046401 E)/0 km	1.40	100.0	10.681060	0.014000 *
95, 562, 563	(32.949536 N, 73.586231 E)/23.56 km	1.26	90.3	7.567004	0.167000
319	(25.736110 N, 69.247760 E)/0 km	1.40	100.0	7.003788	0.262000
136, 112	(31.884300 N, 73.341062 E)/26.33 km	1.24	88.5	4.313667	0.968000
101, 102	(32.322749 N, 72.920919 E)/19.36 km	1.23	88.2	4.103052	0.980000
115	(31.156356 N, 72.751577 E)/0 km	1.26	90.0	4.047079	0.981000
576, 578, 577	(33.474879 N, 74.074645 E)/15.16 km	1.16	83.2	3.684936	0.997000

* significant at the 5% level (*p* < 0.05); ** significant at the 1% level (*p* < 0.01).
